# Renal synovial sarcoma: Considerations for radical nephrectomy- a case report and literature review

**DOI:** 10.1016/j.eucr.2024.102766

**Published:** 2024-06-12

**Authors:** Saeed Montazeri, Mohsen Ayati, Mohammad Reza Nowroozi, Erfan Amini, Seyed Ali Momeni, Tahereh Yousefi, Maryam Azizi, Laleh Sharifi

**Affiliations:** a Uro-Oncology Research Center, Tehran University of Medical Sciences, Tehran, Iran; b Department of Pathology, Tehran University of Medical Sciences, Tehran, Iran; c Department of Pathology, Cancer Institute, Emam Khomeini Hospital Complex, Tehran University of Medical Sciences, Tehran, Iran

**Keywords:** Synovial sarcoma, Renal neoplasms, Primary renal synovial sarcoma

## Abstract

Synovial sarcoma, a rare soft tissue malignancy typically arising from synovial tissue, primarily manifests in the extremities but it may uncommonly present in other locations such as kidneys. Primary renal synovial sarcoma is an uncommon sarcoma with high mortality and recurrence rates. Here, we present a teenage boy with primary renal synovial sarcoma who was referred to our institution.

## 1 Introduction

Synovial sarcoma is a rare mesenchymal malignancy that compromises 5–10 % of all soft tissue sarcomas. The most likely origin of synovial sarcoma is believed to be retrograde differentiation of an undifferentiated mesenchymal cell, often originating from synovial tissue, particularly in proximity to joints in the extremities. However, it can uncommonly present in other anatomical locations such as the thorax, nervous system, prostate, bones, retroperitoneum, and kidneys.^1^

Renal synovial sarcoma is the rarest type of renal sarcoma which is responsible for less than 1 % of all renal masses. It affects both genders of young people ranging from 20 to 50 years. SS18-SSX1, SS18-SSX2, or SS18-SSX4 are identified as specific oncogenes of this tumor. Renal synovial sarcoma is challenging to distinguish from other renal cell carcinomas due to its similar clinical picture to other renal malignancies. Currently, there is no confirmatory diagnosis of synovial sarcoma based on clinical or imaging findings.^2^

In this report, we present one case of primary synovial sarcoma occurring in the kidney who was referred to our clinic.

## 2 Case presentation

**Fig. 1:**
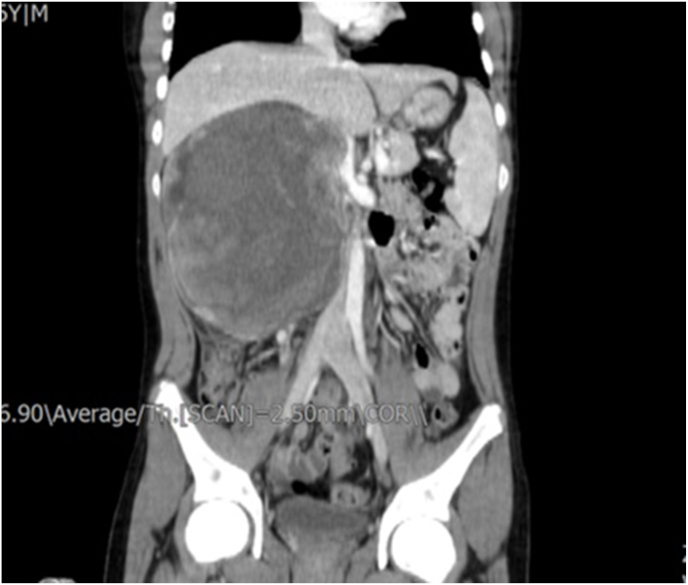
Abdominopelvic CT scan CT scan showed a retroperitoneal mass in size of 250 × 140 × 100 mm.

**Fig. 2:**
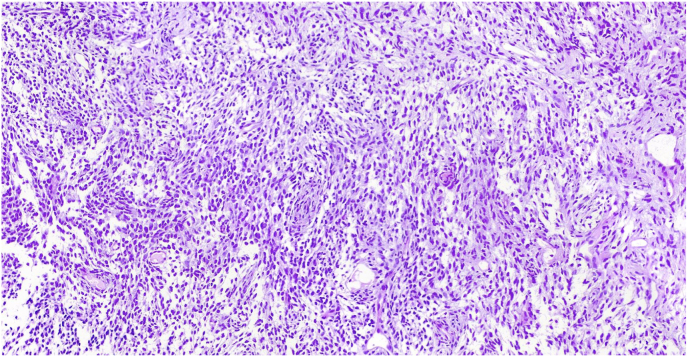
Microscopic view of the resected mass monophasic synovial sarcoma with uniform spindle cells with small nuclei, indistict nucleoli and minute amount of pale eosinophilic cytoplasm. The tumor cells focally arranged in vaguely fascicular pattern.

**Fig. 3:**
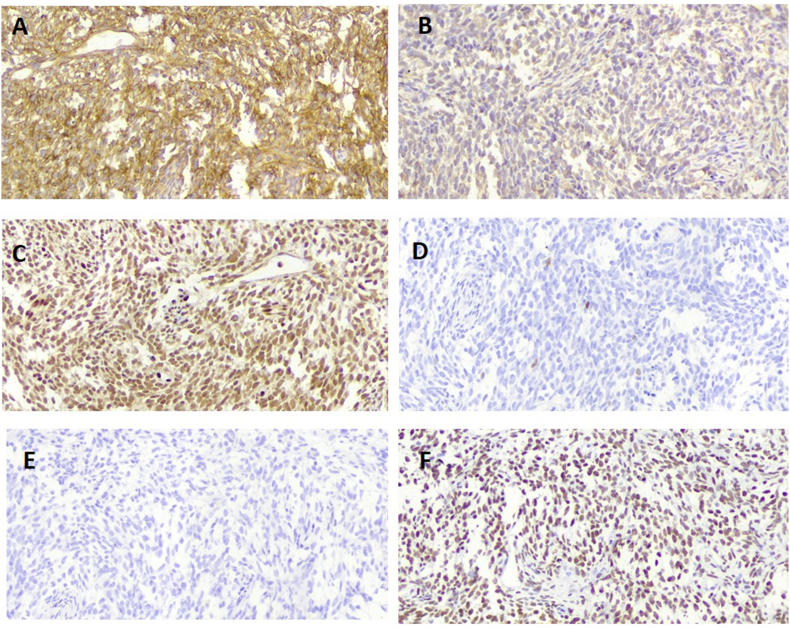
Microscopic view of IHC of the resected mass A: CD99 immunostaining with membranous staining. B: Immunostaining for CK shows patchy positive reaction. C: immunostaining with FLI1 shows diffuse nuclear reaction. D: immunostaining with NKX2.2 shows diffuse nuclear reaction. E: immunostaining with SYN shows diffuse nuclear reaction. F: immunostaining with TLE1 shows diffuse nuclear reaction.

**Fig. 4:**
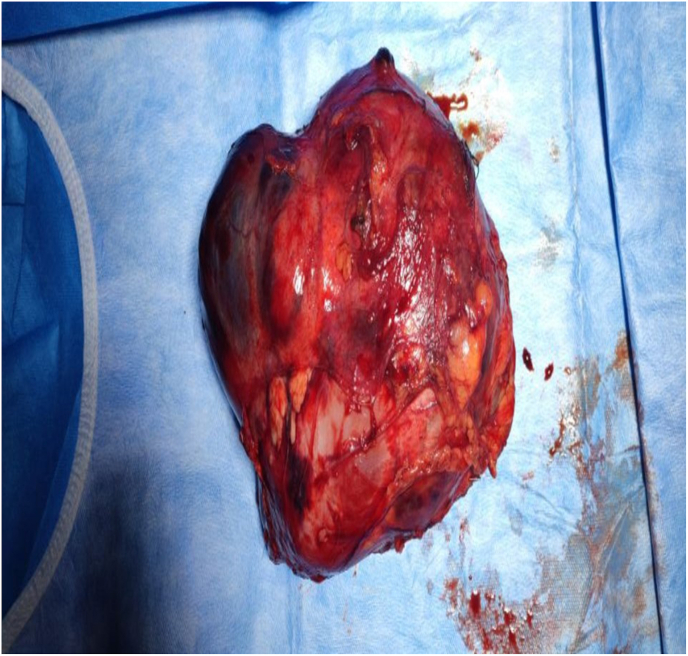
Resected tumor of renal synovial sarcoma.

A 16-year-old boy presenting with right flank pain was referred to our center. Sonographic examination revealed a heteroechoic solid mass with well-defined margins measuring 170 × 150 × 100 mm in the right kidney, originating from the upper renal pole and exerting compressive effects on the liver. Abdominopelvic CT scan demonstrated a retroperitoneal mass measuring 250 × 140 × 100 mm (Fig. 1), while chest CT scan revealed no evidence of metastasis. Renal biopsy was performed, and immunohistochemistry (IHC) testing indicated weak expression of CKAE1/AE3, positive expression of Cyclin D1, TLE1, Bcl2, and FLI1 in some cell sheets, along with 30–40 % expression of Ki67. Morphological examination and IHC findings were consistent with grade 2 monophasic renal synovial sarcoma (Fig. 2, Fig. 3). The patient subsequently underwent radical nephrectomy and lymphadenectomy (Fig. 4). Pathological analysis of the resected tumor confirmed monophasic synovial sarcoma, with lymph nodes reported as free of tumor. The postoperative period was uneventful, and the patient was discharged in good condition after six days. At a 2-month follow-up, he was assessed to be in good clinical condition, and ongoing monitoring is in place to detect any recurrence or metastasis of the disease.

## 3 Discussion

Primary renal synovial sarcoma is an uncommon disease with a mortality rate of 29 % and a recurrence rate of 39.8 %. A review of 163 cases of primary renal synovial sarcoma showed that the monophasic variant was the most frequent histologic subtype (75.4 %) followed by biphasic (14.7 %) and poorly differentiated (9.8 %) subtypes.^1^

Clinical manifestations of renal synovial sarcoma usually include hematuria, abdominal mass, vague pain, local invasion, and distant metastatic disease.^3^

Accurate diagnosis is critical as these tumors have an aggressive and metastatic nature with poor therapeutic outcomes. The diagnosis is problematic because of its rarity and its similar presentation with other renal masses.

Imagings are not distinguishable diagnostic tools for primary renal synovial sarcoma, and diagnosis needs pathological confirmation^4^; therefore, we carried out a preoperative biopsy for our patient. Surgery is the treatment of choice; therefore, we resected the tumor accompanied by lymphadenectomy to decrease the risk of metastasis.

Tumor size and grade play pivotal roles in determining the prognosis of the disease, with radical surgical resection being the preferred treatment. Neoadjuvant chemotherapy is recommended for high-grade and large tumors to reduce mass and facilitate surgery. However, there is limited data demonstrating significant survival improvement with chemotherapy, and the impact of its timing (before or after resection) is uncertain. Furthermore, pre- or post-operative radiation therapy has shown no significant effect on patient survival, although neoadjuvant chemotherapy combined with radiation may offer some benefits.^5^

Available data showed that surgical resection of the renal mass was the most frequent treatment in 95.7 % of patients and chemotherapy as adjuvant treatment was used in 31.3 % of the patients.^1^

Our patient diagnosed with synovial sarcoma originating from the kidney underwent routine diagnostic and treatment approaches. Radical nephrectomy with lymphadenectomy emerged as the primary treatment option for our case.

A systematic review of 96 reports from 2000 to 2018 revealed 34 months overall survival and 25 months disease-free survival for patients with renal synovial sarcoma. They found a significantly lower survival rate for patients with metastasis at diagnosis compared to those without metastasis (6 and 37 months, respectively, P ≤ 0.01). Similarly, patients without recurrence had significant higher survival compared to patients who experienced a recurrence (53 and 24 months, respectively, P ≤ 0.05).^1^

Primary renal synovial sarcoma represents one of the most biologically invasive cancers, posing challenges for conventional treatment methods. Surgical resection alone does not guarantee long-term survival for patients. Unfortunately, alternative therapies such as radiotherapy and chemotherapy have shown limited efficacy. Due to the relative rarity of synovial sarcomas, there is a scarcity of cases available for study. In addition, the follow-up period in most patients was short without complete documentation of disease development.

However, despite these limitations, understanding the molecular mechanisms underlying tumor progression is imperative and should be a focus of future research efforts. Also, more studies are needed to create standard management and surveillance protocols following surgical intervention.

## Funding

No funding was received.

## CRediT authorship contribution statement

**Saeed Montazeri:** Writing – original draft, Investigation. **Mohsen Ayati:** Conceptualization. **Mohammad Reza Nowroozi:** Conceptualization. **Erfan Amini:** Writing – review & editing. **Seyed Ali Momeni:** Investigation. **Tahereh Yousefi:** Investigation. **Maryam Azizi:** Investigation. **Laleh Sharifi:** Writing – original draft, Supervision.

## Declaration of competing interest

The authors declare no conflict of interest.
